# Methodological considerations for observational coding of eating and feeding behaviors in children and their families

**DOI:** 10.1186/s12966-017-0619-3

**Published:** 2017-12-15

**Authors:** Megan H. Pesch, Julie C. Lumeng

**Affiliations:** 1 Division of Developmental and Behavioral Pediatrics, Department of Pediatrics and Communicable Diseases, University of Michigan, 1540 E. Hospital Drive, SPC 5718, Ann Arbor, MI 48109-5718 USA; 2 Division of Developmental and Behavioral Pediatrics, Department of Pediatrics and Communicable Diseases, University of Michigan, 300 North Ingalls Building, SPC 0406, Ann Arbor, MI 48109-0406 USA

**Keywords:** Eating, Feeding, Observational analysis, Video, Behavior, Child, Parent

## Abstract

**Background:**

Behavioral coding of videotaped eating and feeding interactions can provide researchers with rich observational data and unique insights into eating behaviors, food intake, food selection as well as interpersonal and mealtime dynamics of children and their families. Unlike self-report measures of eating and feeding practices, the coding of videotaped eating and feeding behaviors can allow for the quantitative and qualitative examinations of behaviors and practices that participants may not self-report. While this methodology is increasingly more common, behavioral coding protocols and methodology are not widely shared in the literature. This has important implications for validity and reliability of coding schemes across settings. Additional guidance on how to design, implement, code and analyze videotaped eating and feeding behaviors could contribute to advancing the science of behavioral nutrition. The objectives of this narrative review are to review methodology for the design, operationalization, and coding of videotaped behavioral eating and feeding data in children and their families, and to highlight best practices.

**Methods:**

When capturing eating and feeding behaviors through analysis of videotapes, it is important for the study and coding to be hypothesis driven. Study design considerations include how to best capture the target behaviors through selection of a controlled experimental laboratory environment versus home mealtime, duration of video recording, number of observations to achieve reliability across eating episodes, as well as technical issues in video recording and sound quality. Study design must also take into account plans for coding the target behaviors, which may include behavior frequency, duration, categorization or qualitative descriptors. Coding scheme creation and refinement occur through an iterative process. Reliability between coders can be challenging to achieve but is paramount to the scientific rigor of the methodology. Analysis approach is dependent on the how data were coded and collapsed.

**Conclusions:**

Behavioral coding of videotaped eating and feeding behaviors can capture rich data “in-vivo” that is otherwise unobtainable from self-report measures. While data collection and coding are time-intensive the data yielded can be extremely valuable. Additional sharing of methodology and coding schemes around eating and feeding behaviors could advance the science and field.

## Background

Observational measurement of eating and feeding provides a window into behaviors of children and their families that participants may not self-report. This methodology provides the researcher with a unique glimpse into eating behaviors, which can yield rich quantitative and qualitative data. A wide variety of behaviors can be observationally captured. For example, one can examine individual eating behaviors or familial interactions around food, how much food is consumed under certain conditions, environmental factors (where a meal is eaten, how it is served, who is present at the meal), and linguistics of conversation around food. Most studies of eating, feeding and mealtime behaviors have used self-report measures [1, 2], which although less resource intensive, is limited by social desirability bias [3–5]. Observational methodology can capture behaviors of which people are unaware or do not to report, or that are not explicitly asked about. Some prior work [3, 5, 6] has found that self-report and observational measures of eating and feeding behaviors are only weakly correlated. These weak associations may be because these different methodologies are measuring different constructs, with questionnaires measuring parents’ perceptions of behaviors, and videotaped observations measuring objective observations. The weak correlations could also be due to social desirability bias on questionnaires, or the Hawthorne effect [7] that comes with knowing one is being video recorded. Other work [8] has found good correlations between parent report and observed mealtime behaviors, when the constructs from both measures are tightly mapped and highly concordant. It is important to note that self-report measures only capture constructs and behaviors that the questionnaire specifically asks about, and therefore can leave aspects of eating or feeding interactions unquantifiable. Video recorded observational techniques allow for the preservation of a nuanced interaction, which can be reviewed when new questions arise, and re-examined for different facets of behavior. The strengths and limitations of employing observational and self-report methodology for eating and feeding behaviors are summarized in Table 1. The researcher must carefully weigh the strengths and limitations of each methodology, while considering how best to test their hypothesis when choosing their methodological approach. In summary, recognizing that all methods have strengths and limitations, observational methodology captures unique aspects of eating and feeding that enrich our understanding of these behaviors and the contexts in which they occur.

**Table 1:** Advantages and challenges of self-report measures vs. observational coding for measurement of eating and feeding behaviors

	Advantage	Challenge
Self-report measures	• Economical • Low participant burden • Data variables easily created • Able to access beliefs and opinions	• Responses limited by what questions are asked and how they are asked • Social desirability bias • Some groups more prone to responses extremes • Participants may not be aware of the behaviors asked about, or choose not to report. • Difficult to know if reported behaviors reflect “typical” behaviors or attitudes and opinions. • Responses may not be in depth
Observational measures	• Allows for the creation and observation of actual eating interactions. • Can assess multiple participants’ interactions • Able to code quality as well as quantity of behaviors • Able to ask new questions and test new hypotheses not previously tested in the literature • Can review video recordings multiple times to examine behaviors and interactions. • Can control aspects of the environment to test behavioral responses. • Test a hypothesis in a “real life” setting	• Technical challenges • Resource intensive – high cost • Time intensive – data collection, coding and analysis • Higher participant burden • Data variables difficult to generate • Coding is time intensive • Getting reliable is challenging • Hawthorne effect • May not capture “natural” behavior • Limited to the environment captured in the recording. Results may not be generalizable • May lack common coding to compare across studies • Coding schemes are not widely shared or published • Unable to assess attitudes and opinions which may be influencing behavior • Variability in behaviors may not represent “typical” behavior for a participant

Despite the many benefits of observational coding of eating and feeding behaviors, there are potential challenges which deserve consideration in study design and planning. Herein, we will briefly describe some of the challenges. Technical considerations of video recording eating behaviors should not be underestimated. These include ensuring adequate video and audio quality to capture desired behaviors, getting video recording devices to participants along with instructions for set-up, recording and return of devices and data, in addition to storage and management of video files. This can be logistically challenging, and requires a detail oriented staff who can communicate well with participants throughout the process to maintain data quality. Obtaining video recordings of eating and feeding behaviors is more time and resource intensive than questionnaires as it requires more participant time, more involved data management and logistics, and coding of behaviors. Furthermore, as described below, coding behaviors can be challenging and time intensive. As previously mentioned, the Hawthorne effect with regard to participants’ behavior while being video recorded will always be a limitation. However, most of these challenges are surmountable with careful design and flexible thinking and, in our opinion, are outweighed by the quality of the data the can be yielded from observational methodology.

**Fig. 1:**
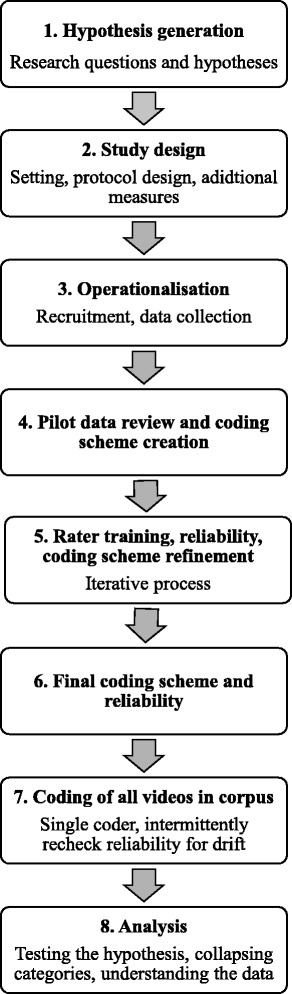
A flowchart of basic steps in the study of observational eating and feeding behaviors

The objectives of this paper are to outline methodological considerations when designing, implementing, coding and analyzing data from a study using observational coding of eating and feeding behaviors in children and their families, and highlight best practices for doing so. An overview of the steps in such a study is presented in Fig. 1.

## Designing the behavioral protocol to be observed

When designing a behavioral protocol to capture eating or feeding behaviors, the authors believe that it is important to start with a hypothesis. What is the behavior or variable that the researcher is most interested in? The answer to this question will guide elements of the behavioral protocol such as whether the video recording should be in a naturalistic environment (i.e., at home) or in a laboratory setting. If, for instance, the researcher is interested in testing if maternal-child food talk varies based on portion size of a meal presented, then researcher may opt to conduct their study in a laboratory based setting in which food can be precisely portioned, and audiovisual equipment can capture the verbal and physical interaction. If, on the other hand, the researcher is interested in examining whether the structure of family meals (e.g., location of the meal, whether the family says a blessing before the meal, if the meal is eaten at a table, etc.) is associated with child weight status, obtaining videos from a naturalistic environment would be more appropriate. Being hypothesis driven will also be important in determining the parameters placed around the video recording – including how the food is presented, types and portions of foods served, length of video recording or food exposure, people present during food consumption, etc. There are many considerations that contribute to careful design of a protocol, which are essential to ensuring that the data obtained will be optimal quality to test the hypothesis.

### The setting

The setting of a protocol to capture eating and feeding behaviors is a fundamental consideration in study design, as the setting may have a strong influence on participants’ behavior [9]. The setting of a protocol can be a naturalistic environment (e.g., in the home or at a restaurant), a laboratory, or a semi-naturalistic environment with controlled elements (e.g., a simulated laboratory restaurant). The advantages and disadvantages to each type of setting, (summarized in Table 2) should be carefully weighed when choosing a study design to best test a hypothesis. Naturalistic settings theoretically capture people exhibiting more naturalistic behaviors [10]. For instance, in a home mealtime recording participants may be more at ease and may be more likely to eat and interact in a more natural manner, allowing for the capture of data that is closer to “real life”. Additional advantages include being able to capture the eating interaction between multiple family members or participants more easily than arranging for a laboratory visit for multiple people. The researcher may also gain insights into the ways in which food is prepared and served, who is present at the meal, and what additional events are going on at the same time (e.g., TV on, people coming in and out of the home, mobile devices present, etc.) [11]. The day-to-day variability of eating in naturalistic settings is another important consideration [12, 13] – (e.g., just because a family orders in pizza one night, or has grandmother over to eat with the family, does not necessarily mean that this is an everyday practice). Additional disadvantages to observations in a naturalistic setting include the lack of experimental control with regard to the ways in which the foods are served or eaten, difficulties in measurement of food intake, and who is present at the mealtime [14]. Technical considerations around video recording are also a major consideration, as sound quality, lighting, movement of the participants during the mealtime out of the view of the camera, and background noise may all interfere with the quality of data captured. Some of these issues can be overcome if a research assistant delivers the video recorder to the participant(s) and instructs the family on its use, retrieving it after the first session or after subsequent sessions [8], however this may come at the expense of social desirability bias, as it is an additional reminder to the participants that they are being observed, and is labor intensive [15]. In addition, it is important to consider that for all individuals present in a video recording for research purposes, informed consent or assent must be obtained [16].

**Table 2:** Advantages and challenges of different settings for the observation of behavioral eating and feeding

Setting	Examples	Advantages	Challenges
Naturalistic	Home mealtimes, restaurants	• Hawthorne effect may be somewhat lessened in a more familiar naturalistic environment • Repeated measures more easily obtained • Provides glimpse into participants’ “real world” behaviors and environment • Many participants can collect data at the same time (can send out multiple video cameras to multiple families)	• Greater potential for lost data • Video and audio quality not assured • Protocol or meals are not standardized and therefore are more variable • Additional participants need to have informed consent completed • Resource intensive • Cannot measure amount of food eaten by each individual
Laboratory	Structured laboratory settings	• Controlled laboratory environment • Environment can be standardized or manipulated to answer specific questions • Video and audio files in control of the research team • Able to measure amount of food consumed • If multiple laboratory settings exist, can run several participants at once	• Hawthorne effect heightened • May be difficult for participants to travel to the location • Technical issues (video camera, equipment failure) • Difficult to obtain repeated measures
Semi-naturalistic	Laboratories set up as restaurants, or dining room/kitchen spaces	• Hawthorne effect theoretically lessened • Controlled laboratory environment • Environment can be standardized or manipulated to answer specific questions • Video and audio files in control of the research team • Able to measure amount of food consumed	• Resource intensive – few facilities exist • Technical challenges with regard to hidden cameras, microphones • May be difficult for participants to travel to the location • Scheduling of facilities (only one family/individual can use the facility at a time) • Difficult to obtain repeated measures

In a laboratory setting, the researcher has much more control over the experimental design and implementation [17]. The manner in which the protocol is set up can be standardized, including the physical environment, the ways in which food is presented (e.g., buffet style, family style, individualized portions), who is present for the protocol, and length of time the participants are exposed to a food. In addition, the type and amount of food served can be carefully controlled and standardized across participants. The researcher can also manipulate the independent variables, and can more accurately measure intake [17]. Technical issues such as video and sound quality are easily controlled, and the researcher can troubleshoot any issues that arise in the moment. However, as in all data collection relying on video recorded data, the laboratory setting is not exempt from technical challenges (e.g., battery or power issues, video recorded malfunctioning, etc.). A challenge of the laboratory environment is that participants may interact with each other and food differently in an unfamiliar environment [18]. A final setting, the semi-naturalistic environment, consists of a replication of a naturalistic environment in a laboratory setting that allows for tighter control of experimental and technical elements, but may put the participants at ease. Examples of these types of arrangements include a laboratory designed like a kitchen and dining room [19] or a simulated fast-food restaurant [20], equipped with “hidden” cameras. While the participants know about the presence of the cameras, they may eat and interact in a more naturalistic manner due to the camera not being in front of them on a tripod as a constant reminder.

For all settings, the impact of social desirability bias and the Hawthorne effect [7] on participants’ behavior due to the presence of a video camera is difficult to gauge–participants may behave differently because they are aware of being observed, to avoid embarrassment, or to “look good” to the researcher, which may differ from their typical behavior. Observations in naturalistic or semi-naturalistic environments may theoretically lessen this effect, but cannot eliminate it.

When choosing a setting, it is important for the researcher to weigh the strengths and weaknesses of each setting type, as mentioned above. The authors feel that a guiding question can be whether or not the hypothesis requires testing of a tightly controlled exposure (e.g., time, food type, amount consumed, presentation, etc.). If the hypothesis requires a tightly controlled exposure, then a laboratory setting is likely best. However, if the research design does not require a tightly controlled exposure and the researcher wishes to capture more “typical” behavior, then a naturalistic setting should be chosen. Lastly, the researcher may choose a semi-naturalistic setting if they want to both control for an exposure as well as capture more typical behavior.

### The set-up

Regardless of the setting of the video recording, the authors suggest that it is important to explain the purpose of the protocol to participants in a scripted and standardized manner, carefully veiling the purpose of the study and the hypothesis being tested. The participants should not be aware of the specific goals of the study or the specific behaviors being observed, to avoid biasing their behavior. Special attention needs to be paid so that the script is not leading, but also meets the ethical obligation to inform the participants about the study. For instance, if the amount of food consumed in a particular condition is the outcome to be measured, it is important to avoid implying to the participants that they are expected to eat the food (which could serve to increase their consumption). Rather, instructing the participants to “eat as much or as little as you’d like”, allows for more flexibility in the participants’ behavior. In a home mealtime recording, the same considerations apply when explaining the purpose of the video recording to the participants. The authors have found that explanations can be as vague as, “we want to learn how families do dinner”.

### Technical issues

When video recording, the frame of the video should to be standardized so that the desired behavior is captured [21]. The authors have found that there are many considerations for the researcher that should be outlined explicitly in the protocol to optimize quality data collection. The researcher must consider if they want just an individual in the frame, an entire group or family or a dyad. If multiple people are present, do they all need to be facing the camera, or is it acceptable for some people to have their backs to the camera? If there is an index participant who is the focus of the study, can other people be eating with him or her, and does the index participant need to be in the center of the frame? Is it important to have the food being eaten in view of the camera, and if so, does this include the plate/dish/wrapper from which the individuals are eating, and/or any larger serving containers? Does the view of the participants need to be unobstructed?

The length of the recording should be specified [22]. Does the researcher need a fixed length of time for the eating segment (this is easy to do in a laboratory setting), or should the participants be allowed to decide when they are “done”? In a naturalistic setting, clear instructions should be provided to the participants with regard to how to set up the video recorder, when to turn it on or off and how to set up the video frame. In a laboratory setting, these details should be articulated in a standardized written protocol. Files of the video recordings, regardless of setting, should be labeled with the participant’s unique identifying number after the file is obtained.

Lighting and sound quality can vary greatly in naturalistic settings [21] and, in the author’s experience, are more easily controlled in a laboratory setting. There are additional challenges of returning the video camera, or digital files containing the video recording to the research team when recordings are completed by participants in naturalistic settings. Associated costs and logistics must be considered in study planning.

### Special considerations for naturalistic settings

There are some additional special considerations for observed eating or feeding behaviors in naturalistic settings. Given variability across days, it is important to consider how many events or meals are needed to achieve acceptable reliability across meals. In addition, the number of meals necessary to achieve reliability across meals depends on the behavior being evaluated. It is also important to obtain a sense from the participants as to whether or not the meal or eating interaction was “typical” for the family. This is important to know given the inherent variability of naturalistic settings (e.g., child is unwell or has a tantrum, unexpected mealtime visitor, etc.) and the common desire for studying “typical” behavior.

After the meal, it is often necessary to document who was present and what was served, as this may not be apparent when reviewing the video recordings. The authors have found that this can be done via phone call by a research assistant, or self-recorded by the participant. This information should be collected in a standardized manner across mealtimes or eating events and participants.

Lastly, the authors have encountered instances in which interactions or behaviors captured on video cause concern for the safety of participants, including concerns for child abuse or neglect. Prior to implementing a protocol and collecting data, it is important to have a plan in place regarding how to report these concerns to the appropriate agency to protect the well-being of participants. In the United States, researchers are Mandated Reporters [23], meaning that the law requires researchers to report their suspicions of child abuse or neglect to Children’s Protective Services. The laws and agencies may vary in different countries.

## Coding

Coding is a rigorous, systematic and often iterative process that consists of identifying a target behavior or event, and the best way to capture it from video recordings [10]. It is important to consider the potential coding approach during study design [10], and then revisit the feasibility of this approach through review of videos prior to development of the coding scheme. If an event or behavior does not occur in the video recordings as frequently as the researcher had hypothesized (or occurs in nearly every video), the researcher would be prudent to reconsider the approach prior to starting to code.

Questions that need to be answered early in this process to inform the development of a coding strategy include: which individuals (e.g., an individual, a family, a dyadic interaction?) and/or what events (e.g., food being served, mobile device use while eating, conversation, bites of food) are being coded [24]? If events or behaviors are contingent upon one another (e.g., child compliance with maternal prompts to take a bite), multiple behaviors will need to be coded in relation to one another in time.

### Coding approaches: Time and frequency considerations

The time interval for coding must be carefully selected. Videos can be coded as a whole, in intervals or as event based. The selection of coding interval depends on the level of detail that the researcher wishes to capture. For instance, to capture general information about a family mealtime, a researcher may choose to code a video in its entirety for simple binary or categorical variables (e.g., in what room of the house did the meal take place? Which family members were present at the meal? Did the mother sit down at the table? Was the TV audible)? More descriptive questions can be answered as well, (e.g., on a scale of 1–5, how oppositional was the child during the meal?) However, if a researcher desires to know more granular information about the meal or eating event, they may wish to use frequency-based, duration or interval coding.

Frequency or event-based coding records the occurrence of each event in a specific period. This coding approach is best used for discrete behaviors that are easily counted (i.e., each time a participant picks up his/her fork and puts food in his/her mouth), as well as shorter observational periods, as frequencies can be labor intensive to capture. If the hypothesis seeks to examine the temporal relationship of one event or behavior to another, it is important to also capture the timing of each event.

Duration coding captures the absolute value or percent of the time that a behavior occurs during an observation period (e.g., minutes in which the child is seated at the table during the meal). Duration coding can be used to capture the percentage of time that an individual is engaged in a specific behavior (e.g., percentage of the protocol that a mother was engaged with her mobile device). This type of coding is best used for behaviors that last more than a few seconds and that occur repeatedly throughout a meal or protocol.

Lastly, interval coding is an alternative approach that can alleviate some of the challenges of frequency or duration coding [10]. In this method, the researcher examines whether a behavior or event occurs at all in multiple smaller time intervals during the observation. For instance, a researcher may code whether or not a participant took a bite of food within each 10 s window of a 4-min video observation. This can provide an estimate for frequency and also duration.

### Coding schemes and guides

Coding schemes can be created anew to answer specific questions or previously developed coding schemes may be applied [24]. While many prior studies have coded behavioral aspects of eating and feeding behaviors, few have published their coding manuals in detail. This may be due to word count limitations in print journals (a factor that is less pressing in the age of primarily digital publication), as well as authors’ desires to protect their intellectual property. If a coding scheme is not published, authors will often provide it upon request. The authors argue that in order for the science eating and feeding behavior observations to advance swiftly, more authors should share their coding methodology using open access platforms. Existing coding schemes that capture mealtime and/or eating behaviors are presented in Table 3. These coding schemes may need to be modified depending on the hypotheses being tested [24]. Chorney et al. [24], have published a detailed guide to developing and refining coding schemes, which is a helpful resource.

**Table 3:** Eating and feeding behavior coding schemes for children over 12 months of age, and their families

Coding scheme name (if applicable)	Authors	Target measures	Coded behaviors or constructs	Coding scheme published	Comments
The ABC Mealtime Coding System	Fiese et al., 2007 [29]	Dimensions of mealtime behavior	Action-oriented behaviors Behavior control behaviors Meal-oriented communication Positive communication Critical communication	No	Micro-systems coding scheme that captures detailed interactions of caregivers and children at mealtime.
Behavior of Eating and Activity for Children’s Health Evaluation System (BEACHES)	McKenzie et al., 1991 [13]	Dimensions of children’s physical activity, eating behaviors and related environmental elements	Environment Physical location Activity level Eating Behavior Interactor Antecedents Prompted event Child response Consequences Events receiving consequences	No	Can be applied in many different environments. Codes antecedents as well as child eating (or physical activity) behavior changes (increase or decrease). Environmental variables contextualize behaviors. Original manuscript employed 60 min observations. Gives extensive information about the eating context, but offers limited descriptions of eating behaviors itself.
Bob and Tom’s Method of Assessing Nutrition (BATMAN)	Klesges et al., 1983 [30]	Child eating behavior and related physical and social environment variables	Child’s eating environment Child behavior Family member interactions with child (encouragement, discouragement, modeling, prompting etc.) Child’s response to interaction	Yes	Original form uses partial interval time sampling – in 10 s windows the child’s behavior as well as the person interacting with the child and the manner of interaction are coded. Live coding is implemented.
Dyadic Interaction Nomenclature for Eating (DINE)	Stark et al., 2000 [31]	Parent and child mealtime behaviors	Parent behaviors: Direct command, indirect command, coax, reinforce, parent talk, physical prompt, feed. Child behaviors: Non-compliance to direct commands, refuse/complaints about food, requests for food, child talk, away from table/food. Child eating	No	All behaviors are coded from video on an occurrence/nonoccurrence basis in 10 s intervals, with the exception of bites, which were counted per 10 s interval.
Feeding Behavior Coding System	Hughes et al., 2007 [32]	Child care provider’s feeding behaviors	Nature of the feeding directive (authoritative, authoritarian, indulgent, uninvolved), the frequency of directive and the food group to which the directive was targets (fruit, vegetable, entree, starch).	No	22-item checklist measure of capturing observed feeding behaviors among child care providers, developed from the Caregiver Feeding Styles Questionnaire [33].
Family Mealtime Q-Sort	Kiser et al., 2010 [34]	Domains of family mealtimes	Positive Tone Meaningful Conversation Clear Plan Disruptions Parenting Style Involvement	Yes	54 item measure describing mealtime characteristics, occurrences and practices on a 9 point scale.
Family Mealtime Coding System	Haycraft and Blisset, 2008 [5]	Parental feeding practices	Pressure to eat Physical prompt to eat Restriction of food intake Use of incentive/conditions	No	Based on subscales of the Child Feeding Questionnaire [35].
The Feeding Scale	Chatoor et al., 1997 [36]	Domains of dyadic feeding	Dyadic reciprocity Dyadic conflict Talk and distraction Struggle for control Maternal non-contingency	No	Developed to evaluate feeding disorders in infants, has been validated in children up to 3 years old.
Iowa Family Interaction Rating Scale	Melby et al., 1998 [37]	Dyadic, family-level interpersonal and dynamics	Interpersonal and family-level (11 domains) and parent-level dynamics (10 domains)	Yes	Similar to the McMaster Mealtime Interaction Coding System (MICS) in that this coding scheme captures macro-level interpersonal family dynamics are coded. Can be applied to a mealtime context. [38]
McMaster Mealtime Interaction Coding System (MICS)	Dickstein et al., 1994 [39]	Family functioning at mealtime.	Task accomplishment Communication Affective interaction Interpersonal involvement Behavior control Roles Overall family functioning	No	Family mealtime observed in the home environment. Each dimension scored on a 7-point scale, from 1 “very healthy” to 7 “unhealthy”. Widely used, with good validity. Focus is more on family dynamics rather than food consumption.
Mealtime Observation Form	Benson and Munoz, 2004 [40]	Structural characteristics of a meal	Length of meal Number of adult and children present How the meal is served Where the meal takes place How many times the child and parent leave the table Types of foods served Is TV/radio/music on/off Does child get second helpings Beverage of child Is dessert served Parents response to child’s picky eating behavior	No	Widely used form in child eating and feeding studies [41–43]. Straightforward coding scheme that is likely easy to apply reliably.
Mealtime Observation Schedule (MOS)	Sanders and Le Gris, 1989 [44], Sanders et al., 1993 [45]	Parental feeding practices and children’s problem and appropriate feeding behaviors	17 categories of child-feeding behaviors (11 categories of disruptive mealtime behavior and 6 categories of appropriate mealtime behavior) 14 categories of parent behavior (aversive behavior and 8 categories of non-aversive behavior.	Partial	Derived from the Family Observation Schedule [46]. Coded in 10 s time blocks during a 20-min observation period. Measures derived from the MOS include % of overall melt during which particular behaviors are exhibited (i.e. % of intervals with a disruptive feeding behavior present).
Parent Modeling of Eating Behaviors (PARM-O)	Palfreyman, Haycraft, Meyer, 2015 [8]	Parental role modeling of eating behaviors	Verbal modeling Behavioral Modelling Unintentional Modeling	No	Developed along with the self-report questionnaire version for parent’s report of their role modeling.
Responsiveness to Child Feeding Cues Scale	Hodges et al. [47]	Maternal responsiveness to child feeding cues	Caregiver general responsiveness during feeding Child feeding cues Caregiver responsiveness to child feeding cues	Partial	Detailed coding scheme that allows for the micro and global analyses of dyadic feeding interaction from early infancy to toddlerhood. Codes child’s hunger and fullness cues separately from caregiver’s responsiveness to those cues. Child feeding cues are additionally divided into Early, Active and Late cues. This coding scheme would likely require a graduate level coder to apply given its detail.
Revised BATMAN	Koivisto et al., 1994 [48]	Child eating behavior and related physical and social environment variables	Additional child behavior categories: Positive food statements Negative food statements Neutral food statements Statements from children about their own eating Additional parent behavior categories: Positive statements about food Negative statements about food Neutral statements about food General nonfood statements Positive statements about child eating	Yes	A revised version of the BATMAN for video recording, with additional categories added for both child and parent behaviors.
–	Cooke et al., 1997 [49]	Temporal patterns of food intake	Food types consumed throughout a meal	No	Laboratory meal protocol. Videos coded in 10 s intervals for foods consumed throughout the meal under two conditions to assess temporal patterns of intake in subjects with eating disorders.
–	Cousins et al., 1990 [50]	Characteristics of food served and consumed at mealtime.	Foods served during meal Method of preparation Number of helpings Estimated portion sizes Amount food eaten	No	Live coding employed to measure characteristics of food served and eaten at a mealtime. Form used to count and record events around food preparation and consumption. Could be applied to video recorded eating interactions if camera angle captured preparation, serving and consumption.
–	Cousins et al., 1990 [50]	Food related interactions between parent and child	For each interaction the following are coded: 1) Time it occurred 2) Persons involved 3) Parental control strategies 4) Child’s response	No	Adapted from prior works [51, 52] for mealtime interactions. Originally employed using live coding. Captures dyadic interactions around food (parent action and child response), but does not capture quality of interaction.
–	Fisher et al., 2013 [53]	Self-served portion size and energy intake in a controlled experimental setting	Number of entrée spoonfuls served Self-served portion size (g)	No	Controlled laboratory setting where pasta was served. Systematically varied the amount available for self-serving and size of serving spoon. Number of spoonfuls served were recorded. Simple and straightforward coding scheme.
–	Iannotti, O’Brien and Spillman, 1994 [54]	Encouragement and discouragements of child eating	Initiator of interaction The food involved If the interaction was to encourage, discourage or exchange the particular food The structure of the interaction Whether a nutritional or other rationale was used to induce compliance Child’s response to the statement	No	Captures the social influences on a child’s eating behaviors including the food involved, and the type of command. This coding scheme is unique in that it captures whether a caregiver gives a nutritional rationale for the command. Also captures child’s response. Unclear if this coding scheme is would be easily applied as the definitions of the codes are not widely available, however the authors suspect that it might be quite nuanced.
–	Pesch et al., 2016 [55]	Home mealtime practices	Child eating at a kitchen/dining room Table (Y vs N) TV audible (Y vs N) Mother sits at the table to eat or drink during the meal (Y vs N)	No	Simple dichotomous variables capturing limited mealtime practices.
–	Pesch et al., 2016 [56]	Affective tone of mother’s statements to restrict child eating	Statements categorized as having positive or negative affect	No	Characterizes mother’s tone and affect around restrictive feeding interactions. Descriptions of tonality may be difficult to interpret and apply reliably.
–	Power et al., 2015 [57]	Maternal verbalization and non-verbal behavior during mealtime	Maternal responsive and non-responsive feeding practices.	No	Event coding scheme developed from an adaptation of prior work [51, 58].

The authors have found that there is great value in developing a new coding scheme which can capture novel behaviors or events. To begin, the researcher should watch many video observations to get a sense of how frequent the behavior/event is and whether it is best captured qualitatively, categorically, or as a count. These observations will guide the time segment considerations in coding previously described. While watching the videos, the researcher should take notes to describe the behavior in a specific and detailed manner, defining the specific parameters of the behavior or event, and differentiating between categories if applicable. It is equally important to describe what does not qualify as meeting the specified parameters of a behavior (e.g., a sip of water does not count as a bite of food). This level of specificity will improve the likelihood that the codes will be applied in a reliable manner. Coding categories can be binary (e.g., did an event happen? Yes, vs No) or categorical (e.g., the child took a small, medium or large serving of food). An excessive number of categories will make reliability difficult to achieve, but an insufficient number of categories may lose important nuance in the data. After drafting a coding scheme, the researcher should apply it to several videos and refine it. Explicit directions should be included for the application of the coding scheme including where to access and store data, how many passes should be used to code, if all behaviors or events should be coded in the same pass or one at a time, etc.

### Refining a coding scheme and reliability

Once the initial draft of a coding scheme is created, two to three coders should independently apply it to a preselected random sample of video observations to achieve familiarity with the coding scheme. The authors usually start with 5 observations. Coders then meet to compare their codes and discuss disagreements or points of confusion, which may lead to the modification of the coding scheme. This iterative process will continue, with the application and subsequent modification of the coding scheme, until the researcher feels that the final coding scheme has been developed. On all codes where disagreements occurred, the team must come to a consensus. Alternatively, one researcher can code all videos with a coding scheme, and a second coder can be brought in later to establish inter-rater reliability. This practice is sometimes necessary given limited resources or the complexity of a coding scheme, however can be higher risk if reliability cannot be established, and the original coding scheme needs to be modified which would result in re-coding of videos.

It is important to note that reliability can be difficult to establish at times, even for seemingly simply codes. Each coder brings with them their own set of experience and biases, which influence how they interpret and code behaviors. Even in simple coding approaches, such as frequency or event based coding, there can often be challenges in establishing reliability between raters, especially for rare events or if events are easily overlooked. Difficulty in establishing reliability may be due to a variety of factors such as a lack of explicit detail in the coding guide or need for more simplistic coding conceptualization, or a need for training sessions and discussion of disagreements in the coders. A resource recommended by the authors for improving and troubleshooting reliability issues for videotaped behavioral observations is Haidet et al., 2009 [22].

Once it seems that the coders are similarly interpreting the coding scheme, all coders should independently apply the coding scheme to 20–30% of video segments, again randomly sampled. Inter-rater reliability should be calculated based on a Cohen’s kappa [22] (for categorical codes) or an intraclass correlation coefficient (for continuous codes, such a Likert scales or counts), which should exceed 0.7 or 0.8, respectively, for each code [25]. If reliability is achieved, then the remainder of the videos may be coded by a single coder. If raters are not reliable for all codes, disagreements should be discussed, the coding scheme refined, and the process of independently applying the new coding scheme to new videos must be repeated. After reliability is established for all codes, the videos used for “training” and reliability establishment must be coded with the final coding scheme. Furthermore, it is important to periodically check inter-rater reliability to ensure no significant drift has occurred during coding of the corpus.

When selecting coders, it is best to train individuals who are blind to the research hypotheses, to avoid unintentional bias. It is important to consider the complexity and nuance of the behavior being coded, and whether a specific coder skill set or background is needed [24] (e.g. choosing between undergraduate students vs. doctoral students). It is also recommended to train more coders than the researcher anticipates needing to guard against staffing changes impeding progress.

Finally, the authors have found that it is useful to pause after coding about 25–30% of the corpus of videos and review the frequencies of behaviors being coded. Is this a rare event that is worth capturing at all? If so, should the definition be changed? Should the code be abandoned or the time segment broadened (e.g. does this behavior occur in this 5 s segment versus does this behavior ever happen at all in this video)? There is often a need to balance the labor and time that goes into coding an uncommon event with the value of that event data to the greater project.

## Analyses

Once the data have been painstakingly coded, the next step is to analyze the data, which largely depends on how the data were coded [26]. While original hypotheses will drive the study design and therefore the analysis plan, there is still some flexibility. It is helpful to first examine the frequencies, means, ranges, and standard deviations of codes. For counts of behaviors, one can examine a summary score of all counts in a video segment (e.g., number of bites during a meal) latency to first event (e.g. time to first bite), or change in event rate over time (e.g., bite rate at the beginning of the meal vs middle vs the end). If interval coding was performed (i.e., whether a behavior was present (vs. not) in each 5 s interval), the proportion of time engaged in that behavior can be calculated. If certain behaviors or events occur infrequently, then code categories can be created (e.g., did the child ask for second helpings >5 times, 4–5 times, 1–3 times, or not at all). Categories can be collapsed (e.g., did the child ask for second helpings >3 times, 1–3 times, or never) or changed into whole video coding (e.g., did the child ever as for second helpings? Yes vs. no). Macro-level behavioral coding can be applied using a simple spreadsheet to capture codes, and video playing software. Micro-level coding such as time-window sequential analysis [27] will require additional training and use of coding software, such as Observer XT [28]. A detailed resource on analyzing data from behavioral observation is the text by Suen and Ary, 2014 [26].

## Best practice highlights

The authors outline below what they consider to be best practices for observational coding of eating and feeding behaviors in children and their families.

- **Start with a hypothesis.** The researcher should be sure to understand the question they are asking and how they want to test their hypothesis. This will guide selection of the setting.
- **Select the setting based on the hypothesis**. If the researcher wants to examine a behavioral response to a specific exposure, then a tightly controlled laboratory experiment may be the best fit. Whereas if the observer wants to examine behaviors in a naturalistic setting, for instance differences in foods served when families eat at the kitchen table vs. other locations, then a naturalistic setting would be a better fi.
- **Carefully plan the protocol using an extensively detailed guide.** The authors recommend writing out explicit step by step instructions that are detailed so that each protocol can be set up as similarity as possibly to improve scientific rigor. It is important to specify the “who, what, where, when” and especially the “how” of a behavioral protocol.
- **Pilot the protocol.** As unforeseen events can occur even with the most seemingly straightforward protocols, it is important to pilot and refine the protocol before involving participants. This will highlight areas of challenge that can be avoided or minimized when collecting valuable participant data.
- **Double check video and audio recording devices.** Prior to an encounter with a participant, it is paramount to ensure the recording devices are working properly to minimize technical issues that may impair data collection.
- **Review data once collection has started, pilot a coding scheme and adjust protocol as necessary.** Once data collection has started, it is important to review the video recordings as well as develop and pilot a coding scheme. This will help the researcher understand if their protocol is successfully testing their hypothesis, or if changes need to be made before data collection is complete.
- **Train raters on reliably coding scheme application through an iterative process.** This will likely involve training of the coder as well as multiple revisions of the coding scheme to add more detail, specificity around the concepts described
- **Trouble-shoot reliability issues.** Reliability should be calculated at the beginning of coding as well as periodically throughout the coding process to protect against drift. When difficulties in reliability present it is important to examine the coding scheme (e.g. definitions of codes may need to be specified or categories may need to be collapsed), the coding approach (e.g. frequency based vs. binary coding, or widening a time interval if too narrow). It is important to examine both the coding scheme, as well as its application by the coder. Some coders may not have the necessary insights, observation skills or attention to detail to apply all coding schemes, therefore it behooves the researcher to train more coders than they think are necessary.
- **Analyze the data to test the hypothesis.** At the end of the long road of observational data collection and coding, the analysis allows for the opportunity to test the hypothesis. Examine univariate data (e.g. frequencies, means, standard deviations) first. Categories with low frequencies may need to be collapsed into larger categories.

## Conclusions

Observational coding of eating and feeding behaviors is a resource and time intensive process, but can yield invaluable data. Researchers can minimize the challenges, and maximize the benefits of this methodology by carefully considering their hypotheses prior to data collection, and designing their protocol and coding approach to address their questions. Additional sharing of methodology and coding schemes around eating and feeding behaviors could advance the science and field.
